# A prospective non-randomized two-centre study of patients with passive faecal incontinence after birth trauma and patients with soiling after anal surgery, treated by elastomer implants versus rectal irrigation

**DOI:** 10.1007/s00384-012-1468-9

**Published:** 2012-05-11

**Authors:** S. J. van der Hagen, W. van der Meer, P. B. Soeters, C. G. Baeten, W. G. van Gemert

**Affiliations:** 1 Department of Surgery, University Hospital of Maastricht, Boerhaavestraat 1, Stadskanaal, 9501 HE the Netherlands; 2 NPBC (Nederlands Proctologisch Bekkenbodem Centrum), Assen, the Netherlands; 3 Refaja Hospital, Stadskanaal, the Netherlands

**Keywords:** Passive faecal incontinence, Birth trauma, Anal surgery, Elastomer implants

## Abstract

**Aim:**

This study is a prospective evaluation of patients with passive faecal incontinence and patients with soiling treated by elastomer implants and rectal irrigation.

**Patients and methods:**

Patients with passive faecal incontinence after birth trauma resulting from a defect of the internal sphincter and patients with soiling after previous anal surgery were included. All patients underwent endo-anal ultrasound, magnetic resonance imaging, and anal manometry. The patients with passive faecal incontinence were initially treated by anal sphincter exercises and biofeedback therapy during half a year. The patients completed incontinence scores, a quality of life questionnaire, and a 2-week diary card.

**Results:**

The elastomer group consisted of 30 males and 45 females with a mean age of 53 years (25–77). The rectal irrigation group consisted of 32 males and 43 females with a mean age of 50 years (25–74). At 6 months follow-up, 30 patients with soiling of the rectal irrigation group and only nine patients of the elastomer group were completely cured (*p* = 0.02). Only three patients with passive faecal incontinence were cured in the rectal irrigation group and none in the elastomer group. Three distal migrations of elastomer implants required removal at follow-up.

**Conclusions:**

After patients had performed anal sphincter exercises, no clear improvement of passive faecal incontinence was obtained by elastomer implants or rectal irrigation. However, rectal irrigation is far more effective than elastomer implants in patients with soiling.

## Introduction

### Passive faecal incontinence

Passive faecal incontinence is caused by incomplete evacuation of stools and/or an insufficient anal sphincter. Some of these patients passively loose stools predominantly during walking. These faecal losses are not severe but have nevertheless a major negative impact on quality of life. Diets to solidify the faeces and sphincter exercise are frequently prescribed but do not always have satisfactory effects. The internal anal sphincter (IAS) provides most of the resting anal pressure and is the main muscle responsible for the prevention of anal leakage [1, 2]. Degeneration of the IAS is presumed to be the cause of passive faecal incontinence in the elderly [3]. Complex operations such as the dynamic graciloplasty or the newer artificial bowel sphincter provide an elevation in resting pressure. These operations are most appropriate in case of major sphincter disruption. They require considerable expertise to achieve good results and are associated with a relatively high complication rate [4, 5]. Most patients have only a partial defect of the internal anal sphincter. Bulking agents like elastomer implants may have beneficial effects in patients with passive faecal incontinence [6, 7].

### Soiling

Soiling is only mentioned in papers dealing with ano-rectal diseases but never discussed on its own merit. Nevertheless, patients with soiling have “a leakage problem”, which presents often after defecation, but they are most of the time continent for flatus and liquids. These patients loose “brown fluids” and suffer from anal dermatitis and itching. Anorectal manometry is within the normal range [8]. Soiling is caused by insufficient clearing of the anal canal after normal defecation. This occurs when sticky faeces stays behind in an anal canal that is anatomically disrupted. The anatomical abnormality may be present in patients with haemorrhoids or in patients who underwent anal surgery resulting in scar tissue or key-hole defects. The scar lesion (key-hole defect) is one important aspect of soiling; however, there are also patients with a key-hole defect without faecal soiling. Since there is also no effective surgical therapy for soiling, injection of bulking agents like PTQ implants may alleviate symptoms by promoting complete evacuation of faeces by restoring the anal canal [8, 9].

Complete evacuation of faeces by enemas or rectal irrigation after defecation appears to be an alternative in conservative treatment. This therapy is described in studies for soiling and retentive encopresis in children [10, 11]. Gosselink et al. [12] reported beneficial effects of retrograde colonic irrigation in patients with bowel disorders and faecal incontinence and soiling.

### Study design

In a prospective non-randomized two-centre study we compared the effectiveness of elastomer Implants or rectal irrigation with 500 ml water after defecation in a group of patients with soiling and another group with (true) passive faecal incontinence due to a defect of the internal sphincter after birth trauma.

## Patients and methods

Between January 2007 and August 2009, 150 patients with faecal soiling or passive faecal incontinence were recruited in two clinical centres. In both centres, the same scoring system and work-up was used for soiling and faecal incontinence by a multidisciplinary pelvic floor team. In one clinical centre, the physician assistant is experienced in the treatment by elastomer implants and in the other centre by rectal and colonic irrigation. The study protocol was approved by the ethical committee. Written informed consent was obtained before entering the study.

### Patients with passive faecal incontinence

Female patients with passive faecal incontinence after birth trauma were included. For inclusion and exclusion criteria, see Table 1. A defect of at least the internal sphincter was confirmed by endo-anal ultrasound and MRI. A dynamic MRI was performed to exclude rectal prolapse and rectoceles. Patients completed the Cleveland Clinic Florida-Faecal Incontinence score (CCF-FI) and the faecal incontinence quality of life score (FIQL). They were initially treated in both centres by anal sphincter exercises and biofeedback therapy by a certified pelvic floor physiologist during half a year. After this training, all patients with a Cleveland Clinic Florida-Faecal Incontinence score (CCF-FI) ≤ 8 were examined for the purpose of this study.

**Table 1:** Patients with passive faecal incontinence

Inclusion criteria
Birth-trauma in history
Passive faecal incontinence
(CCF-FI) ≤ 8 after anal sphincter exercises and biofeedback therapy
Defect of the IAS
Exclusion criteria
Immunosuppression
Prior elastomer implants
Defect of the pudendal nerve
Inflammatory bowel disease
Spinal cord injury
Acute inflammation, infection, malignancy, or post radiation
Current pregnancy or planned further vaginal deliveries
Rectal prolapse
Rectocele > 1 cm
Low anterior resection
Age younger than 18

### Patients with soiling

Patients with soiling after previous anal surgery were examined for the purpose of this study. Patients suffering from itching and fluid loss with or without perianal dermatitis (local reaction and irritation of the dermis) and no true passive faecal incontinence were included. For inclusion and exclusion criteria, see Table 2. They underwent an endo-anal ultrasound examination and static MRI scanning to exclude lesions of the external sphincter. Anal manometry (a polygraph; Medical Synectics, Stockholm) had to be within the normal range (resting pressure, >40 mmHg/maximum squeeze pressure, >75 mm Hg). Patients with faecal soiling completed the Vaizey incontinence score, KEA quality of life questionnaire score (an EuroQol-5D instrument for faecal incontinence [13]), and a 2-week diary card. The Vaizey incontinence and KEA quality of life score was added because soiling is an important variable in this score system in contrast to the CCF-FI and FIQL [13].

**Table 2:** Patients with faecal soiling

Inclusion criteria
Soiling:
Itching and
Fluid loss and/or
Perianal dermatitis (a local reaction at the anodermal skin resulting in itching)
The results of anal manometry within the normal range
Anal surgery in history
Exclusion criteria
Associated external sphincter defect
Immunosuppression
Haemorrhoids grade IV
Faecal incontinence
Inflammatory bowel disease
Current pregnancy or planned further vaginal deliveries
Rectal prolapse
Rectocele > 1 cm
Low anterior resection
Acute inflammation, infection, malignancy, or post-radiation
Age younger than 18

## Elastomer implants

Between January 2007 and August 2009, all consecutive patients of one centre with faecal soiling and passive faecal incontinence were offered treatment by elastomer (PTQ™) implants. Written informed consent was obtained before starting therapy. PTQ Implants (formerly called Bioplastique™) are solid, irregularly textured, medical grade polydimethylsiloxane elastomer implants suspended in a hydrogel carrier of polyvinylpyrrolidone (PVP or povidone). Injections were performed on an outpatient basis under local anaesthesia. A combination of xylocaine 1 % and marcaine 0.25 % was infiltrated into the skin over the proposed site of injection with the cutaneous entry site >2 cm lateral to the sphincter complex and into the deeper soft tissue structures. Patients were injected in the supine or back position. Injecting needle tips were positioned into the submucosal internal sphincter interface under guidance of a palpating finger. Three 2.5 ml injections were placed at 3, 7, and 11 o’clock in supine position. All procedures were performed under protection of a prophylactic single dose of intravenous metronidazole 500 mg and cefuroxime 1,500 mg. Postoperatively oral antibiotics were continued for 5 days (amoxicillin/clavulane acid 625 mg three times daily) [8]. In the event of treatment failure or partial improvement requiring further elastomer implantations, the re-implantation was not carried out until 4 weeks after the initial procedure.

Adverse effects were defined as moderate (superficial temporary infections) and severe (abscesses needing drainage procedures and removal of the implant).

## Rectal irrigation

In the other centre between January 2007 and April 2009, all consecutive patients with faecal soiling and passive faecal incontinence were offered treatment by rectal irrigation. The nurse practitioner specialized in treatment of faecal incontinence explained the use of 500 ml of water containing bottles (REPROP^®^) for irrigation and trained the patients in using it. Irrigation had to be performed after defecation.

The Cleveland Clinic Florida-Faecal Incontinence score (CCF-FI) and the faecal incontinence quality of life score (FIQL) were repeated 6 months after starting treatment.

## Statistical analysis

At baseline and at 6 months, comparisons between elastomer group and rectal irrigation group were performed with *t* test (or Wilcoxon test as appropriate) for continuous variable and chi-squared test for categorical variables (or Fisher’s exact test as appropriate). A *p* value of <0.05 was considered significant.

## Results

### Patients with faecal incontinence

**Fig. 1:**
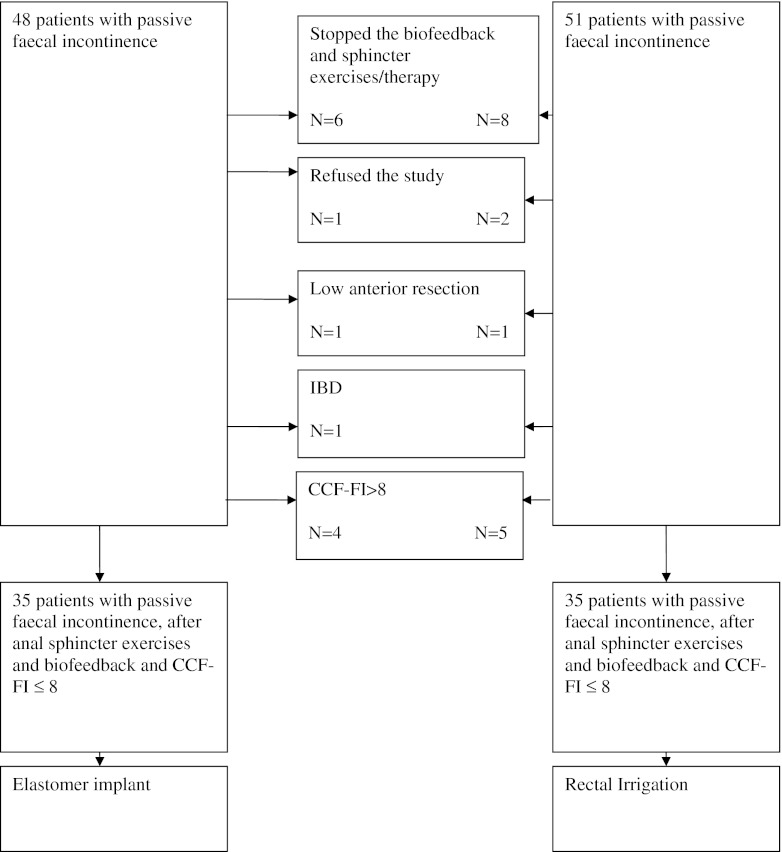
Flow chart of patients with faecal incontinence

Between January 2007 and August 2009, 70 female patients with a mean age of 54 years (range 32–77) with faecal incontinence were included (flowchart, Fig. 1; the patient characteristics and outcome are described in Table 3). All patients returned for follow-up at 6 months. Defects of the internal anal sphincter were observed in 49 (70 %) patients. Degeneration of the internal anal sphincter (mean width <2 mm) was noted in 52 (75 %) patients. In 45 (64 %) patients, a defect of the external sphincter was found on MRI.

**Table 3:** Patients characteristics and outcome; patients with passive faecal incontinence

	Elastomer group *N* = 35		Rectal irrigation group *N* = 35		Statistical analysis	
	Baseline	At 6 months	Baseline	At 6 months	Baseline	At 6 months
Age	55 (32–77)		53 (38–74)		ns	
Patient history:						
Sex(M:F)	0:35		0:35			
Defects of the external anal sphincter	20 (57 %)		25 (71 %)		ns	
Anal manometry:						
Maximum basal pressure (mmHg)	39 (25–51)		41 (29–44)		ns	
Maximum squeeze pressure (mmHg)	69 (57–131)		79 (51–144)		ns	
CCIS-FI	6.2 (4–8)	5.8 (5–8)	6.4 (4–8)	6.2 (0–8)	ns	ns
FIQL:						
Lifestyle	4,5 (3–8)	4.7	4.9	5.0	ns	ns
Coping behaviour	5.3 (3–8)	5.7	4.5	4.0	ns	ns
Depression/self-perception	3.9 (1–6)	4.1	3.8	4.0	ns	ns
Embarrassment	2.1 (1–3)	1.9	2.0	2.0	ns	ns
Faecal incontinence:						
Solid stools (days/week) average	2.2 (0–5)	1.7 (0–3)	2.0 (0–4)	1.9 (0–3)	ns	ns
Liquid stools (days/week) average	1.2 (1–7)	0.9 (0–3)	2.0 (1–7)	1.8 (0–3)	ns	ns
Pads for faecal incontinence Average number/day	0.8 (0–3)	0.9 (0–4)	1.0 (0–3)	0.9 (0–4)	ns	ns

In the elastomer group, in no patient faecal incontinence resolved completely. In the rectal irrigation group, faecal incontinence resolved completely in 3 (9 %) patients. The CCIS-FI score, the average number of days/week of incontinence for solid or liquid stools and the average number of pads used daily did not change significantly after treatment in and between groups. Similarly, no improvement was found in the mean faecal incontinence quality of life score for both groups after 6 months follow-up.

There were no severe adverse effects. In the elastomer group, two infections were observed at follow-up shortly after treatment. Two patients with treatment failure received a second elastomer implant. The implants appeared to be placed subdermally instead of intersphincterically.

In two patients, distal migration of an implant occurred, requiring removal of the implant under spinal anaesthesia after 14 and 18 months of follow-up.

### Patients with soiling

**Fig. 2:**
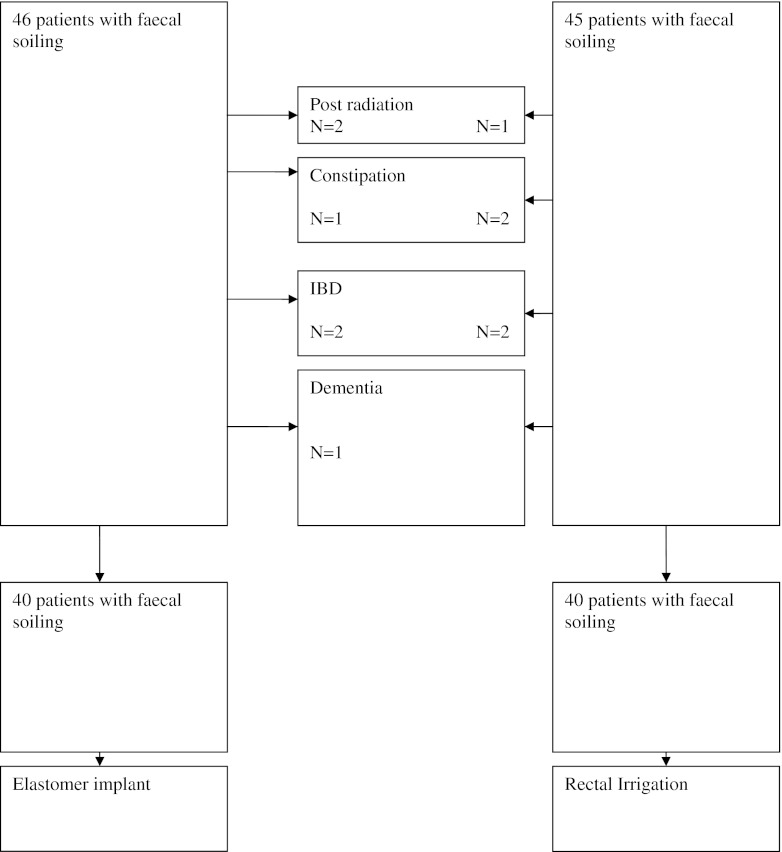
Flow chart of patients with faecal soiling

Between January 2007 and August 2009, 80 patients with a median age of 51 years (range 25–79) with faecal soiling were included (flowchart, Fig. 2; the patient characteristics and outcome are described in Table 4). In the rectal irrigation group, three patients discontinued therapy during follow-up.

**Table 4:** Patients characteristics and outcome; patients with faecal soiling

	Elastomer group		Rectal irrigation group		Statistical analysis	
	*N* = 40	*N* = 40	*N* = 40	*N* = 37		*N* = 37
	Baseline	At 6 months	Baseline	At 6 months	Baseline	At 6 months
Age	51 (28–75)		48 (25–79)		ns	
Sex(M:F)	30:10		32:8		ns	
Patient history						
Haemorrhoidectomy	27		28		ns	
Fistula surgery	6		8		ns	
Other anal surgery	7		4		ns	
Defects of the internal anal sphincter	26 (65 %)		28 (70 %)		ns	
Degeneration of the internal anal sphincter	33 (83 %)		30 (75 %)		ns	
Anal manometry						
Maximum basal pressure (mmHg)	67 (42–86)		64 (40–92)		ns	
Maximum squeeze pressure (mmHg)	161 (202–79)		174 (200–75)		ns	
Outcome						
Patients discontinued therapy		0		3		
Complete response		9 (23 %)		30 (75 %)		0.02
Average daily soiling frequency	2.0 (1–5)	2.1 (0–5)	2.0 (1–7)	0.4 ( 0–2)	ns	0.0001
The average soiling day-time frequency	0.5 ( 0–1)	0.3 (0–1)	1.3 (0–2)	0.45 (0–2)	ns	0.001
The average soiling night-time frequency	0.2 (0–1)	0.2 (0–2)	0.7 (0–1)	0.03 (0–1)	0.06	0.001
The average soiling after stool frequency	2.2 (0–6)	0.5 (0–2)	2.2 (0–7)	0.3 (0–1)	ns	0.001
Patients wearing pads daily	32 (80 %)	20 (50 %)	30 (75 %)	7 (18 %)	ns	0.001
The mean Vaizey-incontinence score	4.2 (0–8)	2.5 (0–6)	4.2 (2–9)	0.9 (0–5)	ns	0.0001
The mean KEA quality of life questionnaire score for faecal incontinence	83 (54–100)	82 (49–97)	81 (53–100)	93 (75–100)	ns	0.0001

The mean basal pressure and the mean squeeze pressure at anal manometry were within the normal range; 66 (40–92) and 168 (75–202) mmHg, respectively.

In the elastomer group, the soiling complaints resolved completely in 9 (23 %) and in the rectal irrigation group 30 (75 %) patients (*p* = 0.02).

The average soiling frequency, the number of patients wearing pads daily and the mean Vaizey incontinence score diminished significantly in the rectal irrigation group compared to the elastomer group after treatment. The mean KEA quality of life questionnaire score for faecal incontinence after treatment improved significantly in the rectal irrigation group compared to the elastomer group.

Endosonography revealed defects of the internal anal sphincter in 54 (68 %) patients. Degeneration of the internal anal sphincter (mean width <2 mm) was noted in 63 (76 %) patients.

There were no severe adverse effects. In the elastomer group, one infection was observed post-treatment at short time follow-up. Two patients received a second elastomer implant because of insufficient bulking at the site of the anal cushions. In one patient, distal migration of an implant occurred that had to be removed under spinal anaesthesia after 20 months follow-up.

## Discussion

Faecal incontinence is a socially and psychologically distressing condition that affects many people. In the United States, 1 in 10 women has faecal incontinence, with 1 in 15 having moderate to severe faecal incontinence [14]. However, approximately 70 % of patients with faecal incontinence do not consult a physician [15]. Therefore, the true prevalence of faecal incontinence and soiling remains unknown and is hard to establish because of underreporting of symptoms by patients, differences in data collection, and different standardized scoring scales [16]. In this study, 16 male patients had soiling complaints for more than 10 years.

There is no established effective therapy for soiling. Mostly, soiling is present in patients having semi-solid faeces, and dietary measures to thicken the faeces is therefore frequently prescribed. In most patients, this therapy is not effective. Patients with a clinical relevant rectocele and prolapse were excluded because the mechanisms underlying faecal incontinence and soiling are different, requiring different therapeutic approaches.

Colorectal surgeons are preferentially applying conservative or minimally invasive treatment for patients with mild passive faecal incontinence and for patients with soiling. The first report of beneficial effects of implanted bulking agents was published in 1993 by Shafik [17]. Several reports followed describing a variable benefit of the use of elastomer implants [6–9, 18–21]. A study in 2007 showed that restoration of the anal canal by elastomer implants also appeared to diminish soiling complaints [8].

The internal anal sphincter (IAS) provides most of the resting anal pressure [22, 23] and is presumed to be the main muscle responsible for the prevention of passive faecal incontinence. Vascular filling in the anal cushions contributes approximately 15–20 % of the resting anal canal pressure [24]. The anal cushions act as a “compliant and comfortable plug” at the anal margin [25]. In contradistinction with passive faecal incontinence, the function and interaction of the anal cushions and the internal sphincter in soiling is still not clear. Anal dysfunction is not the cause of anal soiling. Hoffman et al. [26] found that the mechanism of incontinence in patients with soiling is different from the patients with moderate and severe faecal incontinence in an anal manometric study performed in 170 patients. Felt-Bersma et al. [27] did not find any difference in anorectal function tests between patients with soiling (without faecal incontinence) and the control group, except for the patients with a rectal prolapse. In the present study and a previous study [8], the resting and squeeze pressures of the anal canal in patients with soiling were within the normal range (Table 4).

In our study as well as in other studies, a maximum of 2.5 ml of the elastomer implants (7.5 ml total volume) was used at the sites of the three haemorrhoidal cushions [7, 19]. There are some encouraging earlier studies regarding the treatment of faecal incontinence by intersphincteric elastomer injections [6, 7, 9, 19, 21, 28, 29]. The negative result observed in the present study does not appear to rely on inadequate selection criteria of our patients. All subjects studied had passive faecal incontinence, a CCIS score <8 and none of the patients had a rectocele or an internal or external prolapse. A similar method as in the earlier studies was used to inject the elastomer implants. The elastomer biomaterial was positioned into the intersphincteric space through the anal skin and the external anal sphincter. Tjandra was the first who reported that using ultrasound guidance during injection might lead to better functional results [30]. Maeda et al. reported in a Cochrane review some short-term benefits of ultrasound guidance [31]. But there is still no evidence that ultrasound guidance leads to better outcome. We are familiar with the strategy of the guiding finger. Although impossible to prove on the basis of the present data, we think it is unlikely that the use of the guiding finger instead of guidance by ultrasound is responsible for the disappointing results in this study.

Our results are in agreement however with the study of Siproudhis et al. [32] who in a randomized study could not demonstrate a benefit of intersphincteric elastomer injected by guidance of the finger in the treatment of patients with moderate to severe faecal incontinence. They suggested that the implants might have more benefit in mild faecal incontinence. Soerensen et al. [29] concluded that the benefit of elastomer implants is limited to minor leakage and soiling in a prospective study of 33 patients. In our study, only patients with mild faecal incontinence (Cleveland Clinic Florida-Faecal Incontinence score (CCF-FI) ≤ 8) after birth trauma and without clinical relevant rectoceles and prolapse were included and nevertheless disappointing results were achieved.

Maeda et al. [31] found no objective clinical benefit of bulking agents in patients with fecal incontinence and indicated the necessity of large well-designed trails. Recently, a prospective sham-controlled trial of 206 patients with a variable severity of fecal incontinence was published. The bulking agent dextranomer in stabilised hyaluronic acid was injected and showed in 52 % of the patients a reduction of 50 % or more in incontinence episodes compared to 31 % of the patients with the sham treatment [33]. The authors concluded that the injection of this substance was an effective treatment for faecal incontinence. However, no information was provided regarding the type of incontinence.

No clear benefit of treatment with elastomer implants is demonstrated in the present patient group with passive faecal incontinence after biofeedback therapy and sphincter exercise. Bartlett and Ho [34] reported a prospective study and found that patients who had previously received biofeedback treatment had the poorest results after elastomer implants. No information has been provided regarding the influence of previously received biofeedback treatment and anal sphincter exercise in the other studies.

Antegrade and retrograde colonic irrigation and rectal irrigation are widely used for functional bowel disorders and soiling [11, 12, 35]. This therapy appears to be effective in patients with faecal incontinence and soiling as a result of functional bowel disorders like constipation or a medical history like Hirschsprung’s disease or rectal prolapse. The water removes the remaining faeces in the colon and/or rectum. The present study shows that irrigation of the anal canal in patients with soiling after anal surgery without functional bowel disorders or rectal prolapse is a very effective and simple treatment modality. Minor forms of evacuation like suppositories seem to offer a more convenient way of treatment. There are no data in the literature to prove this. Nevertheless, we expect that complete cleaning of the rectum is responsible for the good results [36]. The long term therapeutic benefit depends on the severity of soiling complaints on the one hand and the therapeutic compliance of the patient on the other hand.

Rectal irrigation is not sufficiently effective in patients with passive faecal incontinence and an anal sphincter defect. Probably, such patients need colonic irrigation to ensure that a larger part of the colon is irrigated.

We can conclude that in this well-selected group of patients, no clear benefit could be demonstrated from elastomer implants or rectal irrigation in patients with mild faecal incontinence after biofeedback therapy and sphincter exercises. But in the group of patients with soiling, rectal irrigation is effective and superior to elastomer implants. The present study confirms the safety of elastomer implants reported in other studies [37].
